# Case Report: Management and follow-up of continuous positive airway pressure in a Fontan patient with obstructive sleep apnea

**DOI:** 10.3389/fped.2026.1887999

**Published:** 2026-08-25

**Authors:** Andrew Ligsay, Colin Crilly, Conor P. O'Halloran, Philip Thrush

**Affiliations:** 1 Division of Pediatric Cardiology, Ann & Robert H. Lurie Children's Hospital, Chicago, IL, United States; 2 Division of Pediatric Pulmonary & Sleep Medicine, Ann & Robert H. Lurie Children's Hospital, Chicago, IL, United States

**Keywords:** CPAP, Fontan, obstructive sleep apnea, screening, ventricular dysfunction

## Abstract

Patients with single ventricle heart disease undergo staged surgical palliation ultimately resulting in total cavopulmonary anastomosis (Fontan procedure), with circulation that relies on passive pulmonary blood flow. In Fontan patients with comorbid obstructive sleep apnea (OSA), previous reports have suggested that continuous positive airway pressure (CPAP) therapy can be detrimental and negatively affect cardiac function in this population. However, we describe a case of an adult Fontan patient with ventricular dysfunction and severe OSA who demonstrated significant improvement his cardiac index following CPAP use. This case highlights a potential subset of patients with Fontan physiology who may derive significant cardiovascular benefit from CPAP therapy.

## 1 Introduction

A total cavopulmonary anastomosis (Fontan) is the current final stage of surgical palliation for patients with single ventricle heart disease. The Fontan off-loads the work of the heart, allows for passive systemic venous blood to return to the pulmonary circulation, and increases systemic oxygenation. Over the past 50 years since the introduction of the Fontan procedure, improvements in surgical technique and medical management of Fontan patients have resulted in better survival outcomes. Consequently, there is now increasing focus on the long-term treatment of associated comorbidities affecting this population.

Obstructive sleep apnea (OSA) is a common disorder characterized by partial or complete upper airway obstruction resulting in disruptions to sleep and/or associated decreases in systemic oxygenation levels compared to baseline. Typical risk factors include obesity, male sex, and older age. OSA has been associated with increased daytime sleepiness as well as cardiovascular comorbidities such as hypertension, arrhythmias, and heart failure. Current literature suggests that 21%–36% of Fontan patients may be at risk for OSA (1–3), and given their significant underlying heart disease, individuals with Fontan circulation and comorbid OSA may be at greater cardiovascular risk compared with the general population.

Management of OSA in Fontan patients presents unique challenges (4). While nocturnal continuous positive airway pressure (CPAP) is the gold-standard therapy for OSA, previous reports have stated that excessive CPAP may negatively affect cardiac output in Fontan patients (3, 5). However, the goal of this report is to highlight a Fontan patient who demonstrated significant cardiovascular benefit from CPAP use, and to also propose a potential timeline for screening and management of OSA in this population.

## 2 Case description

We present the case of a young adult man with hypoplastic right heart syndrome with underlying cardiac anatomy consisting of tricuspid atresia with pulmonary atresia. He initially underwent placement of a Blalock–Taussig–Thomas shunt within the first week of life, conversion to bidirectional Glenn circulation at 9 months of age, and ultimately extracardiac Fontan palliation at 2 years of age. His clinical course was complicated by sick sinus syndrome, prompting epicardial pacemaker placement at 3 years of age. The Fontan fenestration was closed at 8 years of age. At 19 years of age, he underwent cardiac catheterization, which demonstrated a cardiac index (CI) of 2.3 L/min/m^2^, moderately elevated Fontan pressure of 20–21 mmHg, an indexed pulmonary vascular resistance (PVRi) of 3.98 Wood Units (WU), and angiography demonstrating two large venovenous collateral vessels that were coiled. His body mass index (BMI) at the time of this cardiac catheterization was 38.8 kg/m^2^.

Following this procedure, serial echocardiography studies revealed progressive worsening of his left ventricular function; his left ventricular fractional shortening decreased from 40% to 15% over a 2-year period. He also reported worsened snoring, as well as concern for nocturnal apneas and daytime sleepiness.

## 3 Diagnostic assessment, therapeutic intervention, follow-up, and outcomes

**Figure 1:**
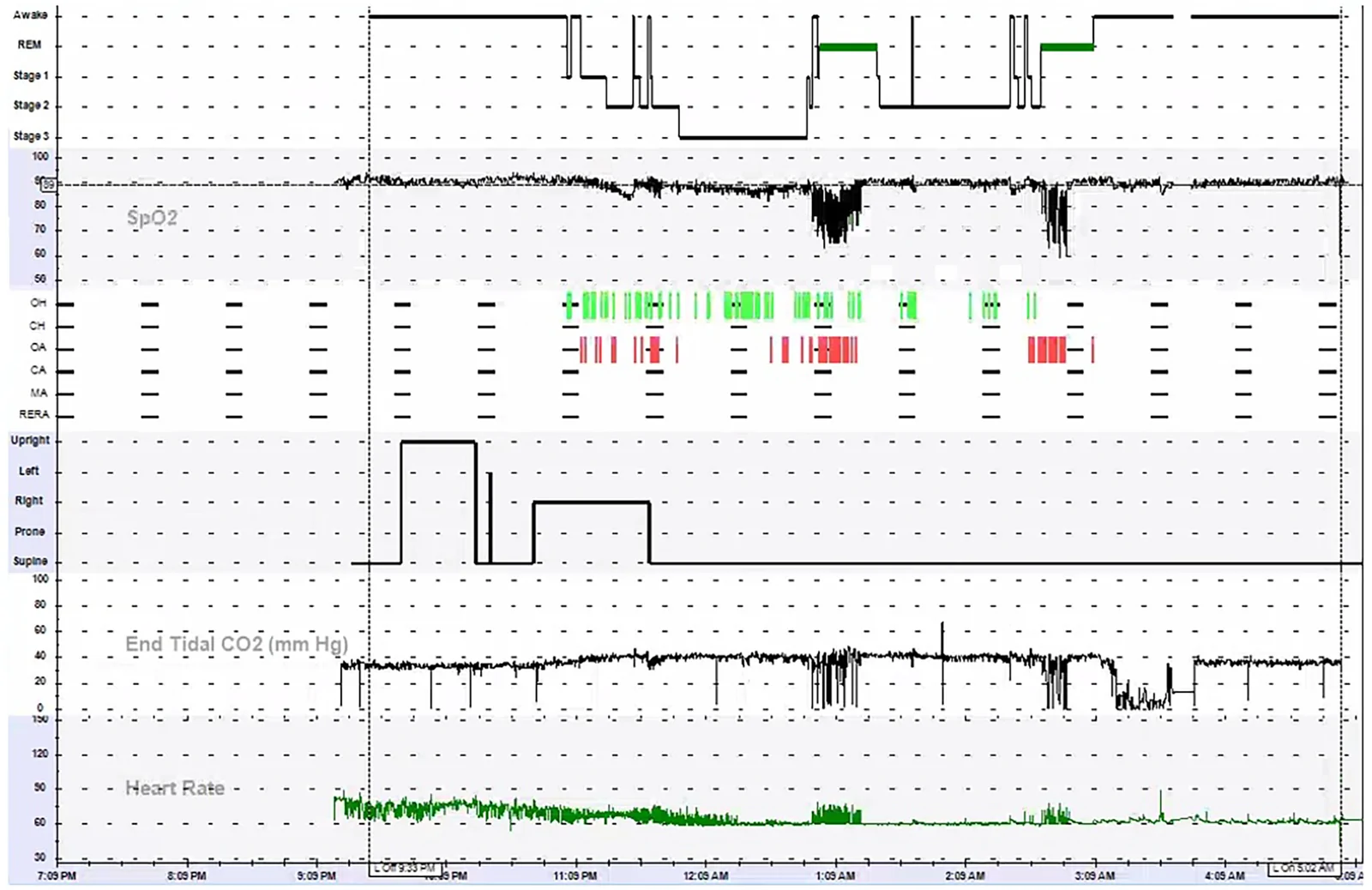
Diagnostic polysomnography hypnogram showing several obstructive hypopneas (row 3, green vertical lines), obstructive apneas (row 3, red vertical lines), and significant hypoxemia (rows 2) during rapid eye movement (REM) stage sleep.

**Figure 2:**
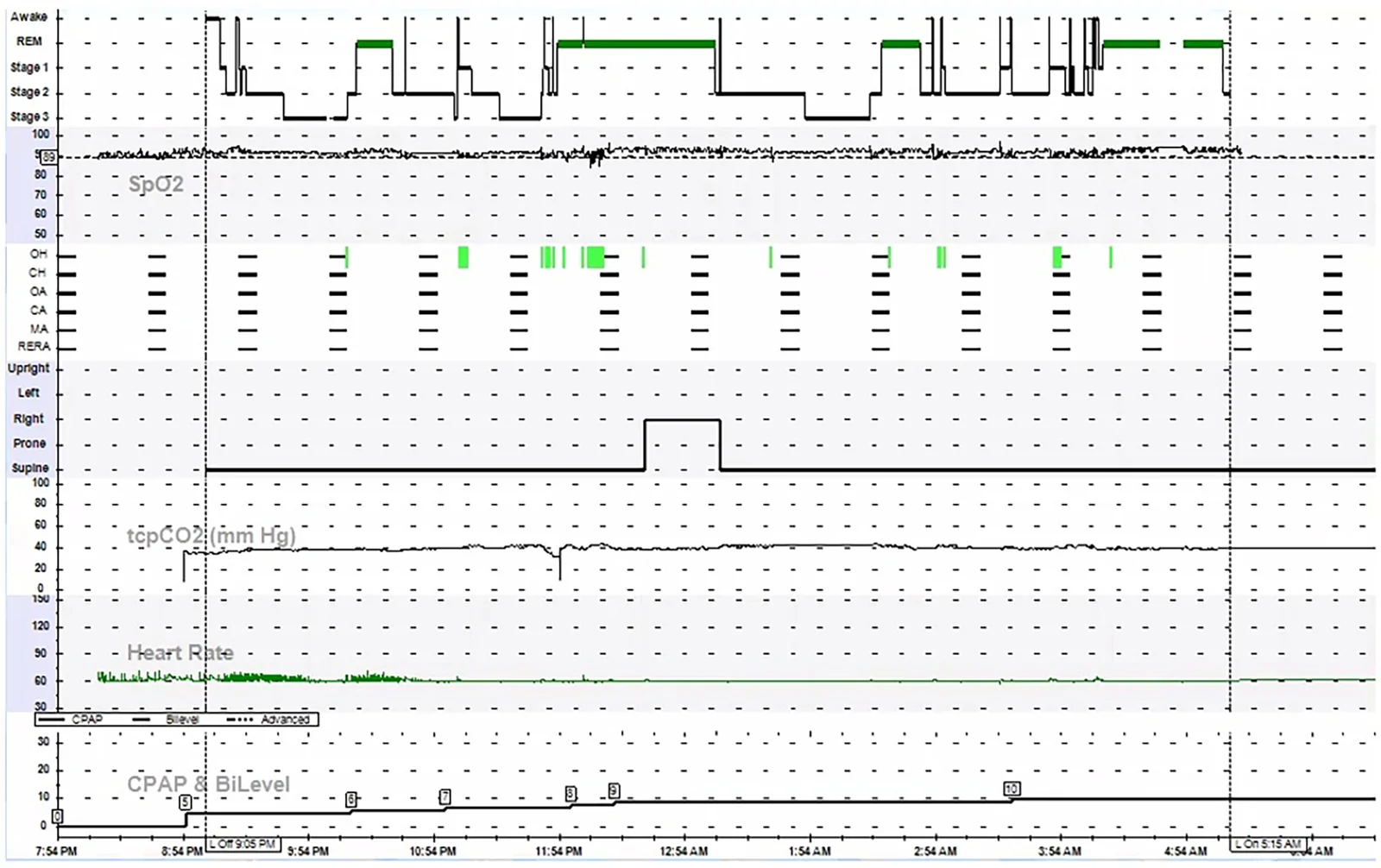
CPAP titration polysomnography hypnogram showing improvements in obstructive hypopneas compared to the prior study (row 3, green vertical lines). Note the resolution of obstructive apneas (row 3, absence of red vertical lines) and hypoxemia (row 2).

At 21 years of age, due to concern for sleep-disordered breathing, the patient established care with sleep medicine. He underwent a diagnostic polysomnography test, which revealed severe obstructive sleep apnea with an apnea–hypopnea index (AHI) of 36.9/h according to the American Academy of Sleep Medicine (AASM) 1A criteria (Figure 1). His baseline nocturnal oxygen saturation was 87%, with SpO_2_ nadir of 59%. End-tidal CO_2_ measurements remained normal (40–49 mmHg) throughout the study. A follow-up CPAP titration polysomnography study showed that his OSA was well controlled at CPAP of +9 and +10 mmHg, with residual AHI of 2.2 and 3.3/h, respectively (Figure 2). SpO_2_ remained greater than 87% at these pressures, and there was no evidence of hypoventilation.

Repeat cardiac catheterization was performed after his titration polysomnography study. At baseline and without CPAP respiratory support, this showed a measured baseline arterial oxygen saturation of 95%, cardiac index of 2.4 L/min/m^2^, Fontan pressure of 17 mmHg, and PVRi of 0.92 WU. To test the safety of CPAP pressures, the patient was placed on CPAP +10 mmHg, which demonstrated slight improvement in his arterial oxygen saturation (97%), an increase in his CI to 3.1 L/min/m^2^ (+29%) with only a minimal change in his Fontan pressure to 18 mmHg, and a decrease in his PVRi to 0.44 WU. His BMI at the time of the procedure remained unchanged compared with his prior cardiac catheterization (38.3 kg/m^2^). His measured hemoglobin was 17.3 g/dL. No changes were made to his pacemaker settings during the procedure.

## 4 Patient perspective

The patient was initiated on CPAP therapy and reported benefit from use at his 3- and 6-month follow-up appointments with sleep medicine. Specifically, he reported feeling more energized and refreshed after nights that he used CPAP therapy. Compliance data from his CPAP machine showed improving compliance (60% of nights; typical therapeutic goal is >80% of nights), with no residual obstructive sleep apnea on therapy (residual AHI of 1.6/h). Lifestyle barriers were identified as his primary challenge to further increase in usage. There were no changes in his weight, cardiac medications, or pacemaker settings during this follow-up period. Of note, a repeat echocardiogram was performed 2 months after his cardiac catheterization, which did not show any changes in ventricular function. Longer-term surveillance of sustained clinical benefit and changes in cardiac function is ongoing.

## 5 Discussion

We report a case of a young adult man with Fontan physiology and ventricular dysfunction who showed significant improvement in both OSA and CI with CPAP use. While CPAP at high pressures has been shown to decrease cardiac output in some individuals with Fontan physiology (3, 5, 6), to our knowledge this is the first case highlighting improvements in OSA and CI with CPAP therapy. This suggests that there may be a subset of patients who respond exceptionally well to CPAP therapy, warranting further exploration.

In univentricular heart disease at baseline and after stage 1 palliation, the functioning ventricle delivers blood in parallel to both the systemic and pulmonary circulations (7). These circulations require mixing of oxygenated and deoxygenated blood, resulting in arterial desaturations, as well as volume overloads the ventricle, which would ultimately lead to ventricular dysfunction and worsening heart failure if not addressed.

However, in the final palliated stage of univentricular heart disease, the Fontan circulation allows blood flow to occur in series. That is, blood is pumped to the systemic circulation for oxygen delivery, then returns passively to the pulmonary circulation via a direct anastomosis of the superior and inferior vena cava into the pulmonary arteries to allow reoxygenation and gas exchange. Once oxygenated, blood continues to flow passively back to the univentricular heart (7). This circulation does not require mixing of oxygenated and deoxygenated blood, and relatively decreases the volume of blood the single ventricle is pumping with each cardiac cycle. Unfortunately, this circulation is unfavorably sensitive to increases in pressure gradients along this pathway, which can then affect the degree of pulmonary blood flow and subsequently cardiac preload and output of the heart. For example, OSA can contribute to reduced cardiac output through impaired gas exchange and resultant increases in pulmonary vascular resistance (PVR), while also increasing afterload on the systemic ventricle through activation of the sympathetic nervous system and renin–angiotensin system (8).

As previously noted, the gold-standard therapy for OSA is treatment with CPAP, which supplies air pressure to overcome the degree of upper airway obstruction and alleviate the effects of sleep-disordered breathing. CPAP, in the treatment of OSA, can improve ventilation and oxygenation, and thus reduce PVR. Furthermore, the increased intrathoracic pressure from CPAP can also reduce the transmural gradient of the left ventricle, thus reducing ventricular afterload. In a left ventricle with depressed systolic function, lowering the afterload can improve stroke volume and cardiac output. Unfortunately, as this therapy increases intrathoracic pressure, higher CPAP pressures may start to hinder passive pulmonary blood flow in the Fontan circulation, at which point it would start to negatively affect cardiac output (3, 5, 6). Therefore, initiation of CPAP in this population should be conducted with care and managed with close follow-up. As with our case, we hypothesize that there is at least a select population of Fontan patients with OSA who can experience significant benefit from CPAP use. This could be because the benefits of adequate gas exchange, reduced PVR, and reduced afterload outweigh the negative effects of increased intrathoracic pressure. In this patient case, the cardiac catheterization data demonstrated both a reduction in the PVR and an increase in the CI directly after administration of CPAP. While his follow-up echocardiogram did not show any sustained changes in cardiac function or remodeling, it is possible that this may be influenced by suboptimal compliance and short-term follow-up imaging.

Limited observational data suggest that patients with single ventricle or Fontan physiology may be at increased risk for OSA before 20-years of age (9). However, while OSA is a known risk factor for cardiovascular disease, there have been no consensus recommendations for the timing of OSA screening in Fontan patients. Moreover, no consensus recommendations for optimal CPAP management in this population have been established as well. Regarding the latter, CPAP titration protocols by cardiac catheterization (5) and echocardiography (3) have both been previously outlined in the literature, but larger studies implementing these protocols have not yet been reported.

In 2019, the American Heart Association released a scientific statement outlining the recommended timeline of cardiovascular surveillance testing in Fontan patients. This statement suggested consideration of cardiac catheterization testing every 10 years beginning in adolescence (10). Given the early risk of OSA in Fontan patients, it may be beneficial to screen for OSA at least prior to cardiac catheterization testing. If a patient with Fontan physiology is found to have significant OSA and requires CPAP, conditional hemodynamic testing with CPAP use can be performed during scheduled cardiac catheterization surveillance.

Overall, this case highlights the complex interplay between respiratory mechanics and cardiac hemodynamics in a patient with Fontan circulation. Moreover, proper management of OSA in Fontan patients has the potential to improve quality-of-life metrics, such as daytime sleepiness, while also mitigating cardiovascular risk factors in this high-risk population. Additional studies are warranted to identify potential exceptional responders to CPAP therapy.

## Data Availability

The original contributions presented in the study are included in the article/Supplementary Material. Further inquiries can be directed to the corresponding author.
